# Association of cardiovascular response to an acute resistance training session with the ACE gene polymorphism in sedentary women: a randomized trial

**DOI:** 10.1186/1471-2261-13-3

**Published:** 2013-01-10

**Authors:** Jéssica Cardoso de Souza, Ramires Alsamir Tibana, Nuno Manuel Frade de Sousa, Vinícius Carolino de Souza, Margô G O Karnikowski, Jonato Prestes, Carmen Silvia Grubert Campbell

**Affiliations:** 1 Graduation Program on Physical Education, Catholic University of Brasilia, Brasilia, Brazil; 2 Graduation Program Inter-unities - Bioengineering, EESC/FMRP/IQSC, USP, Sao Carlos, Brazil; 3 Graduation Program in Medical Sciences, University of Brasilia, Brasilia, Brazil; 4 Physical Education Program, Catholic University of Brasilia, Q.S. 07, Lote 01, EPTC – Bloco G. Zip code: 71966-700 – Aguas Claras – Federal District, Brasilia, Brazil

**Keywords:** Resistance training, Blood pressure, Heart rate, Polymorphism, Angiotensin converting enzyme

## Abstract

**Background:**

The aim of the present study was to verify the effects of an acute resistance training (RT) session and insertion/deletion (I/D) polymorphism of the angiotensin-converting enzyme (ACE) on systolic (SBP), diastolic (DBP) and mean blood pressure (MBP), and heart rate (HR).

**Methods:**

The sample consisted of 27 sedentary women (33.3 ± 8.2 yrs; 69.1 ± 13.8 kg; 1.57 ± 0.05 m; 27.6 ± 5.1 kg/m^2^) divided into two groups according to their polymorphism I/D (DD = 9; II + ID = 18). Volunteers underwent two experimental sessions: RT – an acute session performed with three sets at 60% of one-repetition maximum (1RM) interspersed with 1 minute rest interval between exercises and sets, and a control session (CON) in which they remained seated for 30 minutes in the laboratory. SBP, DBP, MBP and HR were measured before exercise and during one hour every 10 minutes after sessions, in the seated position. A two-way ANOVA for repeated measures with Tukey’s post hoc test was used for the intra and inter-group comparisons.

**Results:**

There were no statistically significant differences on SBP, DBP and MBP after the experimental protocols, and no effect of ACE polymorphism (P > 0.05). However, comparing CON versus exercise effect size values (ES), homozygotic carriers of the allele D presented a drop in SBP which was considered moderate, while in allele I carriers it was small, 30 minutes after exercise. In MBP, homozygotic D carriers exhibited a large ES 20 minutes post-exercise. HR was higher at 10, 20 and 30 minutes after exercise as compared to pre-exercise only for carriers of the I allele (P < 0.05).

**Conclusions:**

Therefore, an acute RT session reduces clinical BP. In addition to this; it seems that ACE polymorphism had some influence on cardiovascular response to exercise.

**Trial Registration:**

RBR-6GDYVZ

## Background

Arterial systemic hypertension is one the most important modifiable causes of cardiovascular morbimortality in the adult population, affecting two to three persons out of every 10, and contributing to myocardial infarction, vascular encephalic accident and chronic kidney disease [1]. According to the statement on coronary disease and vascular encephalic accident from the American Heart Association (2006), it has been estimated that 71,300,000 American adults present one or more types of cardiovascular disease, reaching a proportion of one out of every three individuals with diagnosed hypertension [2].

Similar to type 2 diabetes, obesity, insulin resistance and dyslipidemia, hypertension has a multi-causal nature and the main risk factors are classified as modifiable (lifestyle, smoking, sedentarism, inappropriate nutrition, between others) [3].

Furthermore, hypertension has been frequently associated with genetic factors. For example, polymorphisms of genes are involved in the physiological control of blood pressure (BP) [4]. Some genes have been identified as candidates to explain the variations in BP phenotypes, such as the insertion/deletion (I/D) polymorphism of the angiotensin-converting enzyme (ACE) [5,6].

ACE is a protein produced mainly by the lungs and endothelium, and its main physiological role is to convert angiotensin I into angiotensin II, thus favoring vasoconstriction of blood vessels and increasing BP [7]. The renin-angiotensin-aldosterone system (RAAS) corresponds to a complex endocrine cascade associated with the control of BP and maintenance of hydroeletrolitic balance. Most of RAAS effects are mediated by an active substance called angiotensin II, a product of the conversion from angiotensin I by its catalyst, ACE [7].

Moreover, ACE decreases the formation of bradykinin and nitric oxide, both considered important vasodilators [8]. In this sense, Sayed–Tabatabaei et al. (2006) found that, although individuals are capable of maintaining relatively stable levels of ACE, this enzyme may vary from person to person, indicating a genetic hypothesis to the variation of ACE [8].

The polymorphism corresponding to the insertion (allele - I) or deletion (allele - D) of 287 pairs of bases in the intron 16 of ACE gene has shown an association to cardiac risk factors and vascular disorders. Blanchard et al. (2006) found that homozygotic individuals (deletion) for ACE presented ambulatory hypotension after exercise, while II/ID carriers did not [9].

Regarding lifestyle behavior, the practice of regular exercise has been recommended as a valuable tool to decrease resting BP. Independent of the chronic effects of regular exercise, an acute decrease of BP below resting values may occur after exercise, a physiological phenomena called post-exercise hypotension (PEH) [10]. It has been shown that PEH may last up to 12 to 24 hours [10-12]. Although the hypotensive effects of aerobic exercise have been widely confirmed, the drop in BP after resistance training (RT) has produced controversial results [13]. Tibana et al. [14] showed that an acute submaximal RT session was effective in decreasing systolic (SBP), diastolic (DBP) and mean BP (MBP) during 24h and nighttime. However, another study revealed no changes in SBP and DBP following an acute RT session compared with a non-exercise control day in young, normotensive men and women [15].

To the best of our knowledge, no previous investigations have been designed to analyze the possible influence of ACE gene polymorphism on the clinical response of SBP, DBP and MBP after an acute RT session in sedentary women. Furthermore, the association between the ACE polymorphism and the response of heart rate (HR) to a RT session has not been investigated in this population. Thus, the aim of the present study was to analyze the acute response of BP and HR after a control and RT session, and to verify the possible influence of ACE genotype. The initial hypothesis was that ACE polymorphism could exert some influence on the cardiovascular response to an acute RT session in sedentary women.

## Methods

Initially 41 women were selected for this study. Five discontinued because of difficulties in traveling to the laboratory, four were excluded due to the presence of cardiovascular disease, and five were excluded because they were regular practitioners of exercise. Thus, 27 sedentary women (defined as accruing less than 2h per week of physical activity during the last year) participated in the present study. Each volunteer completed a thorough physical examination, including a medical history, resting electrocardiogram, blood pressure assessment, anthropometric, and orthopaedic evaluation prior to participation in the experimental protocols. The general characteristics of the participants are presented in Table 1. Before participation in the study, volunteers signed a written consent and were informed about the risks and benefits. The present study was approved by the Local Research Ethics Committee for Human Use (Protocol number 376/2010). The inclusion criteria were: pre-menopausal between 25–41 years of age and no regular practice of exercise in the previous 12 months. The adopted exclusion criteria were: use of drugs that could affect cardiovascular response to exercise and the presence of any disease that would compromise their health during the study period.

**Table 1:** **Anthropometric**, **cardiovascular and biochemical variables of the participants according to the angiotensin**-**converting enzyme** (**ACE**)

	**DD** (**N** = **9**)	**II** + **ID** (**N** = **18**)	***P***
Age (years)	30.1 ± 8.3	34.9 ± 7.8	0.15
Body mass (kg)	70.8 ± 15.8	68.3 ± 13.1	0.66
Height (m)	1.61 ± 0.08	1.57 ± 0.05	0.20
Body mass index (kg/m^2^)	27.2 ± 4.3	27.6 ± 5.1	0.85
Waist circumference (cm)	84.6 ± 12.3	83.9 ± 11.5	0.89
Neck circumference (cm)	33.5 ± 2.4	33.9 ± 2.0	0.67
SBP (mm · Hg)	112.9 ± 9.9	114.8 ± 8.4	0.60
DBP (mm · Hg)	73.7 ± 9.5	76.9 ± 6.8	0.32
Glucose (mg/dL)	86.7 ± 5.3	87.1 ± 17.5	0.95
Triglycerides (mg/dL)^†^	87 ± 45.7	92.5 ± 40.8	0.46
HDL-cholesterol (mg/dL)	56.8 ± 13.9	52.5 ± 14.9	0.49
Insulin (μUI/mL) ^†^	11.7 ± 7.6	8.7 ± 5.5	0.56
Glycated hemoglobin (%)	5.3 ± 0.3	5.1 ± 0.5	0.23

^†^ Data presented as median. SBP = Systolic blood pressure; DBP = Diastolic blood pressure.

### Procedures

Volunteers underwent two experimental sessions: RT with 60% of one-repetition maximum (1RM) and a control session without exercise (CON). Experimental sessions were randomized and took place between 19h 30min and 21h 30min. Individuals were advised to avoid exercise and to maintain their normal dietary intake in the 24h before sessions. Additionally, their caffeine and sodium intake were controlled (this was guaranteed by a dietary recall). All procedures were conducted in the laboratory under controlled environmental and temperature conditions.

### Maximal strength testing (1RM)

Individuals completed maximal strength testing two weeks after the adaptation period, which consisted of 8–10 submaximal repetitions of each exercise altering lower and upper limbs. 1RM tests were performed in four distinct days with a minimum of 48h between trials. All tests took place between 19h 30min and 21h 30min with 10 min intervals between exercises tested in the following order: Chest press, front lat pulldown and machine shoulder press (days 1 and 2); machine leg press, leg extension and leg curl (days 3 and 4) (JOHNSON – USA). Exercises were tested in the order that they were placed in the RT session. To obtain reliable strength values, the 1RM trials were performed on separate days, with 72h between them. A high interclass correlation was found between the first and the second 1RM trials, with mean values of R = 0.98 for all exercises.

Following a general warm-up (10 minutes of light treadmill walking), participants performed eight repetitions with 50% of estimated 1RM (according to each individual capacity verified in the adaptation period). After a one-minute rest interval, three repetitions with 70% of 1RM were completed. The subsequent trials were performed for one repetition with progressively heavier weight until the 1RM was determined within three attempts, using 3- to 5-minute rest periods between trials. The range of motion and movement standardization of the exercises was conducted according to the descriptions of Brown and Weir (2001) [16].

### Experimental protocol

The experimental protocol consisted of two trials (control and resistance training) performed in a randomized design with one week interspersed between them. In the control session participants remained resting in the seated position for 30 minutes. The RT session also lasted 30 minutes and consisted of three sets of 10 repetitions for six exercises in the following order: machine leg press, leg extension, leg curl, Chest press, front lat pulldown, machine shoulder press and abdominal crunches, with an intensity fixed at 60% of 1RM (except for abdominal crunches, which were performed with 15–20 repetitions) and a rest interval of one minute between sets and exercises. Additionally, participants were instructed to maintain correct breathing patterns and to avoid the Valsalva maneuver, and room temperature was standardized at 22°C.

### Anthropometric measures

Height was measured by a wall-stadiometer (Sanny, Sao Paulo, Brazil), with a capacity of 2200 mm and precision of 1 mm. Weight was determined on a digital scale (Welmy-W110H, Sao Paulo, Brazil). Circumferences were obtained in triplicate using a nonelastic tape measure, and averaged to determine the final reported circumference. Neck circumference was obtained with the subject sitting with the head in the Frankfort horizontal plane position. A measuring tape was applied around the neck inferior to the laryngeal prominence and perpendicular to the long axis of the neck, while the minimal circumference was measured and recorded to the nearest 0.1 cm. Waist circumference was measured at the level midway between the lower rib margin and the iliac crest.

### Resting biochemical analysis

Blood tests were performed in a central laboratory after at least 12h of fasting. Analytic parameters in plasma were automatically obtained using an EDTA 1-mg/mL tube. Triglycerides, HDL-cholesterol, glucose, glycated hemoglobin and insulin were measured by the following methods: enzymatic CHOP-POD; homogeneous HDL-cholesterol; Hexokinase; HPLC (High-performance liquid chromatography) and Electrochemoluminence, respectively. These data were used to characterize the participants.

### Clinical blood pressure and heart rate

Cardiovascular parameters (SBP, DBP, MBP and HR) were measured with an oscillometric device (Microlife 3AC1-1, Widnau, Switzerland) on the upper right arm according to the guidelines from the American Heart Association (2005) [17]. This method has been validated by the Association for the Advancement of Medical Instrumentation and by the British Hypertension Society. The values of SBP and DBP were used to determine MBP by the following equation: MBP = DBP + [(SBP - DBP)/3]. All resting BP and HR measures were assessed in triplicate (measurements separated by 1 min) with the mean value being used for further analysis. The BP and HR measurements were performed: after 15 minutes of seated rest (Res); and 10 minutes (T10); 20 minutes (T20); 30 minutes (T30); 40 minutes (T40); 50 minutes (T50); and 60 minutes (T60) after the control or exercise session.

### DNA extraction and genotyping

Blood samples were obtained from the antecubital vein by a trained professional. 3 to 5 ml of blood was drawn in vacutainer tubes containing EDTA anti-coagulant. DNA was obtained from peripheral blood leukocytes by using a DNA extraction kit according to the manufacturers recommendations (PureLink Genomic DNA Kits – Invitrogen, Brazil). Extracted DNA was stored at -80°C for subsequent analysis. The insertion(I)/deletion(D) polymorphism in the human ACE gene (rs4646994) was determined by inspection of the electrophoretic profile of polymerase chain-reaction (PCR) products, and performed as described by Marre et al. (1997) with modifications [18]. Either the 490 bp (I allele) or the 190 bp (D allele) products were amplified using primers: 5^′^-CTGCAGACCACTCCCATCCTTTCT-3^′^ and 5^′^-GATGTGGCCATCACATTCGTCAGAT-3^′^, which flank the polymorphic site. Reaction tubes contained 100 ng DNA, 10 mmol/l Tris–HCl pH 8.3, 75 mmol/l KCl, 3.5 mmol/l MgCl2, 0.2 mmol/l dNTP, 20 pmol of each primer, 0.5 μg of purified chicken albumin and 1 U of Taq DNA polymerase (Phoneutria®, Minas Gerais, Brasil) in a final volume of 25 μl. After 1 min of hot start at 80°C and an initial denaturation for 2 min at 94°C, the amplifications were carried out for 30 cycles of 40 s at 94°C, 45 s at 64°C and 50 s at 72°C followed by a final 5 min extension at 72°C. Inspection of DD subjects was carried out using oligonucleotides (5^′^-TGGGACCACAGCGCCCGCCACTAC-3^′^ and 5^′^-TCGCCAGCCCTCCCATGCCCATAA-3^′^) specific to amplify a 335 bp fragment of the insertion sequence. In brief, DNA was amplified for 30 cycles with denaturation at 92°C for 40 s, annealing at 63°C for 40 s, and extension at 72°C for 40 s. All PCR products were separated by electrophoresis on 2% agarose gels containing ethidium bromide at 50 μg/ml, visualized by using CCD camera (Vilber Lourmat®, Eberhardzell, Deutschland), examined using the gel analysis software enclosed (Photo Capt 1D), and confirmed by visual inspection.

### Statistical Analysis

Statistical significance for all variables was fixed at p ≤ 0.05. The Shapiro-Wilk and Levene tests were completed to check the normality and homogeneity of the data, respectively. The sample of 27 women revealed a statistical power of 85%. A two-way ANOVA for repeated measures with Tukey’s post hoc test was used for the intra and inter-group comparisons. Furthermore, one-way ANOVA for repeated measures was used to analyze the cardiovascular response of the whole group, without considering the polymorphism. Independent Student’s *t* test and Mann–Whitney test was used to compare the resting characteristics of both groups. The Statistical Package for the Social Sciences (SPSS, v.19, Chicago, IL) was used. Additionally, the magnitude of differences between alleles and periods was verified by the effect size (ES) according to the recommendations of Cohen (1988) with threshold values of 0.2 (small), 0.6 (moderate), 1.2 (large), and 2.0 (very large) considered [19].

## Results

### Resting parameters

Table 1 presents resting anthropometric, cardiovascular and biochemical parameters of the groups according to ACE polymorphism. There was no difference between groups for age (p = 0.15), height (p = 0.20), body mass (p = 0.66), body mass index (p = 0.85), waist circumference (p = 0.89), neck circumference (p = 0.67), SBP (p = 0.60), DBP (p = 0.32), glucose (p = 0.95), triglycerides (p = 0.46), HDL (p = 0.49), insulin (p = 0.56) and glycated hemoglobin (p = 0.23).

### Acute resistance training session

**Figure 1:**
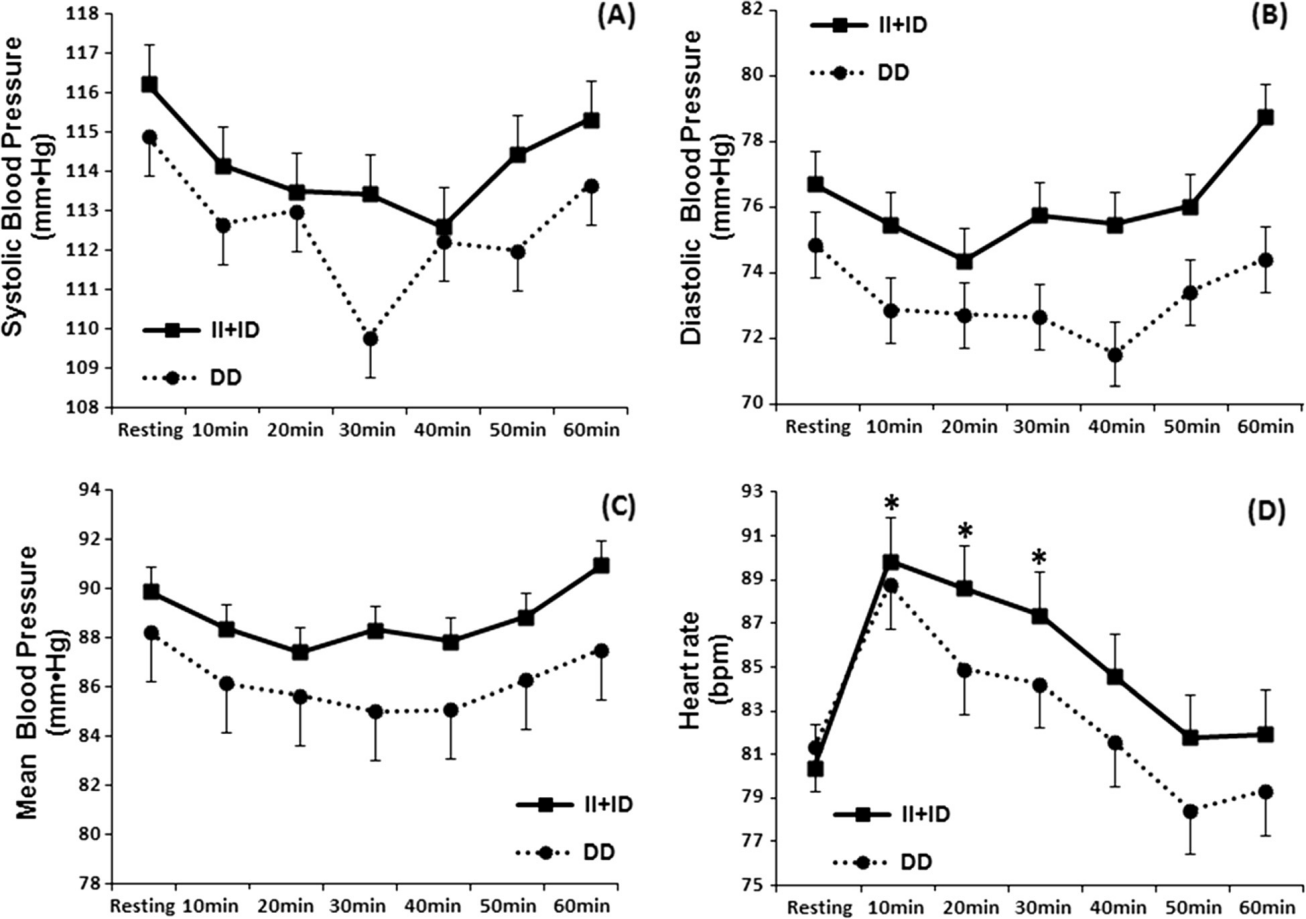
**Systolic (A), diastolic (B) and mean blood pressure (C), and heart rate (D) measured at resting and 10**, **20**, **30**, **40**, **50 and 60 minutes after an acute resistance training session for the groups according to the polymorphism DD and II + ID.** *Statistically significant difference compared with resting (P < 0.05).

Results of the two-way repeated measures ANOVA revealed no significant differences in SBP, DBP and MBP between CON and RT sessions when group averages were compared, as well as between resting and T10, T20, T30, T40, T50 and T60 measures. In addition, this analysis found no differences for group averages between allele I carriers and homozygotic carriers of allele D at any time point (Figure 1).

However, ES analysis comparing CON versus RT revealed that homozygotic carriers of allele D presented a drop in SBP of moderate magnitude, while allele I carriers presented a small magnitude 30 minutes after the RT session. Regarding MBP, both groups presented a decrease of moderate magnitude from minute 10 to 50 and large at minute 60, except homozygotic carries of allele D, which exhibited a large ES 20 minutes after the RT. Moreover, in the whole group analysis, without considering the polymorphism, ES values were small (Table 2). Comparison of resting values with post-exercise values revealed small magnitudes and were not presented in Table 2.

**Table 2:** **Results from effect size** (**ES**) **analysis**

**Systolic blood pressure**	**10min.**	**20min.**	**30min.**	**40min.**	**50min.**	**60min.**
DD (RT vs. CON)						
ES	0.15	0.18	0.67	0.13	0.05	0.23
II + ID (RT vs. CON)						
ES	0.19	0.30	0.26	0.34	0.34	0.26
All (RT vs. CON)						
ES	0.03	0.12	0.24	0.12	0.02	0.09
**Diastolic blood pressure**						
DD (RT vs. CON)						
ES	0.13	0.01	0.21	0.30	0.05	0.29
II + ID (RT vs. CON)						
ES	0.32	0.38	0.22	0.42	0.34	0.01
All (RT vs. CON)						
ES	0.05	0.10	0.08	0.31	0.09	0.07
**Mean blood pressure**						
DD (RT vs. CON)						
ES	1.15	1.31	1.04	1.05	1.16	1.28
II + ID (RT vs. CON)						
ES	1.09	0.98	1.08	1.03	1.14	1.39
All (RT vs. CON)						
ES	0.04	0.11	0.21	0.27	0.07	0.01

RT = acute resistance training session; CON = control session; I = insertion allele; D = deletion allele.

HR values were significantly higher after the RT session at T10 (p = 0.04), T20 (p = 0.03) and T30 (p = 0.03) as compared with resting, only for the II + ID group (Figure 1).

**Figure 2:**
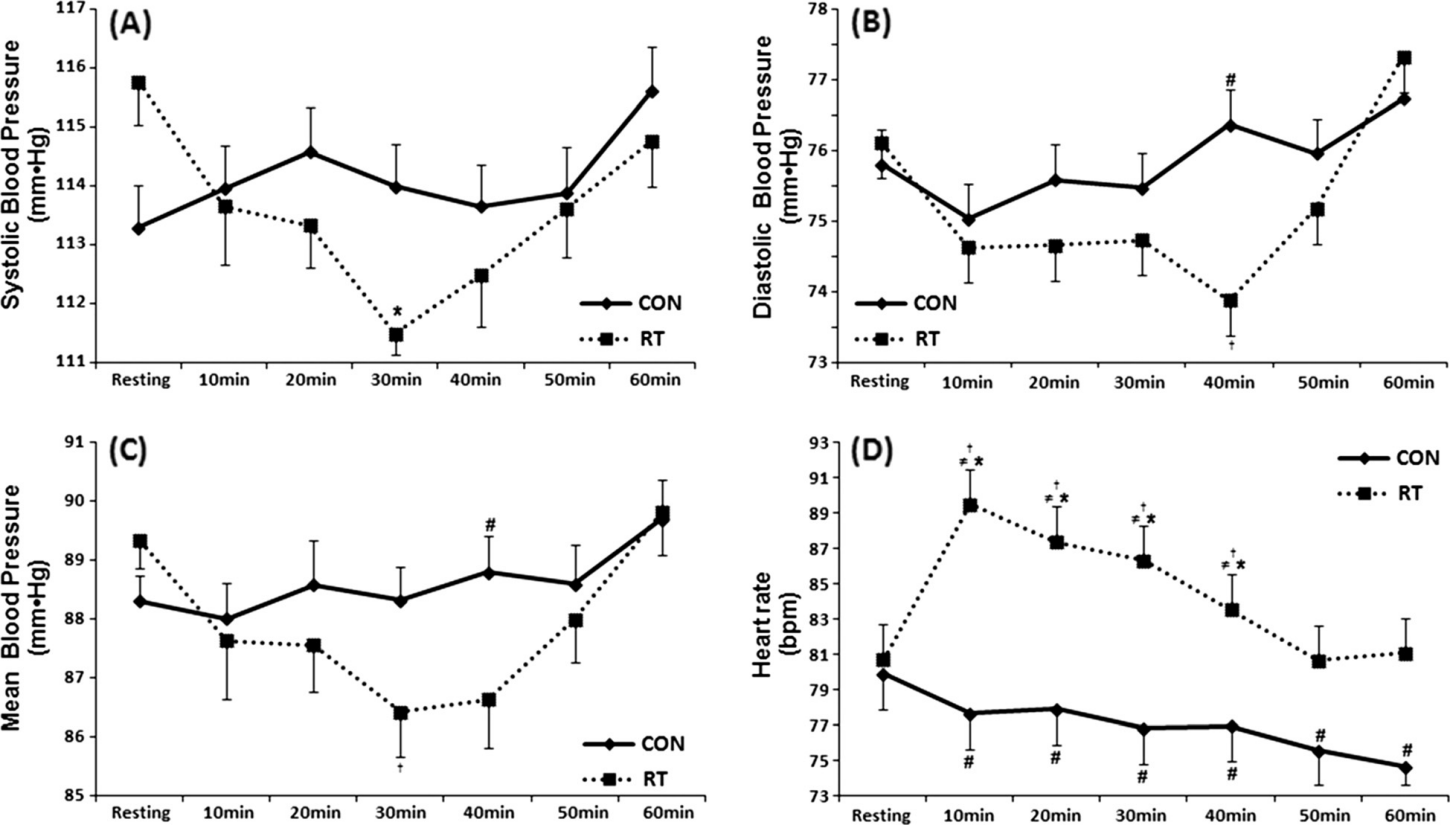
**Systolic (A), diastolic (B) and mean blood pressure (C), and heart rate (D) measured at resting and 10**, **20**, **30**, **40**, **50 and 60 minutes after the control (CON) and acute resistance training session (RT) in all individuals**, **without considering the polymorphism(n** = **27).** *Statistically significant difference compared with resting (P < 0.05); ^#^Compared with the same period of the CON; ^†^Compared with 60 minutes after exercise; ^≠^ Compared with 50 minutes after exercise (P < 0.05).

Figure 2 presents BP and HR data from the whole group, without considering the polymorphism. Results revealed a drop of SBP 30 minutes after the acute RT session as compared with resting. DBP and MBP were lower at minute 40 after the RT session as compared with CON. HR was increased after the RT session from minute 10 to 40 as compared with resting values. HR was increased from minute 10 to 60 in the RT session as compared with CON (p ≤ 0.05).

## Discussion

Results from the present study showed no statistically significant differences of BP response after an acute RT session between different allele carriers of the ACE gene. However, ES showed a moderate magnitude on SBP by comparing the CON versus RT session in homozygotic carriers of the allele D, while allele I carriers presented a small magnitude 30 minutes after exercise. Additionally, homozygotic D carriers presented a large ES on MBP 20 minutes after the RT session. Moreover, HR was higher up to 30 after exercise in the allele I carriers. Whole group analysis, without considering the polymorphism revealed a decrease on SBP 30 minutes after exercise as compared with resting values. In addition to this, DBP and MBP were lower 40 minutes after the acute RT session as compared with the same period of the control session. Therefore, the initial hypothesis was partially confirmed, considering that the ACE polymorphism exerted some effects on the cardiovascular response to an acute RT session in sedentary women. Finally, to the best of our knowledge no previous research has been designed to test this specific hypothesis.

Therefore, two important aspects should be considered: 1) whole group analysis revealed a drop in BP (PEH) and 2) considering the polymorphism, there was no statistically significant PEH, while ES BP values were of higher magnitude in some periods for DD, even with a reduced number of participants in this group as compared with allele I carriers. Thus, ACE gene polymorphism exerted some effect on the acute cardiovascular response to a RT session.

Whelton et al. (2002) found that even less expressive drops in BP, such as 3 to 5 mm·Hg, contribute to a decrease of 8-14% of acute myocardial infarction episodes, 5 to 9% of risk for coronary disease, and 4 to 7% in the general causes of mortality [20]. For example, the drop in SBP 30 minutes after exercise was ~5 mm·Hg for the DD group and ~2.5 mm·Hg for the allele I carriers. The decrease in DBP was ~4.5 mm·Hg for DD carriers versus ~1.5 mm·Hg for the allele I carriers 40 minutes after the acute RT session. These results reinforce the importance of using ES for some biological variables with clinical relevance, especially under situations where conventional statistics reveals no differences.

Busjahn et al. (1997) investigated the influence of ACE gene polymorphism on cardiac dimension and ACE activity in 132 twins, 91 monozygotic and 41 dizygotic. The study revealed that DD carriers presented a higher dimension of posterior cardiac wall and ACE circulating activity, independent of resting BP [21]. Another study showed that, apart from the circulating levels of ACE, the activity of ACE from the heart was higher for DD carriers, while this phenomenon is associated with a higher incidence of cardiovascular disorders [22]. Considering these phenotypic differences between different allele carriers of the ACE gene, it is important to investigate the effect of the ACE polymorphism on the cardiovascular response to exercise.

In response to aerobic exercise, normotensive individuals presented a PEH of 8 to 10 mm·Hg for SBP and 3 to 5 mm·Hg for DBP. Alternatively, in hypertensive individuals this decrease was of 18 to 20 mm·Hg for SBP and of 7 to 9 mm·Hg for DBP [23]. These data reinforce the idea that hypertensive individuals or those with any type of cardiovascular disorder could present a higher magnitude of decrease in BP after exercise as compared with normotensive controls [24]. Thus, the superior magnitude of reduction in BP verified by the ES in the DD group could be associated to the greater prevalence of cardiovascular disorders, inherent to this polymorphism. Similarly, Pescatello et al. (2007) observed a PEH at 40% of VO_2max_ after a cycloergometer exercise session in stage I hypertensive individuals, only in homozygotic allele D carriers [5]. It is important to consider that, similar to the present study, resting values of BP were not different between the distinct alleles.

Alternatively, Santana et al. (2011) found a reduction of BP after a maximal incremental cycloergometer exercise test only for allele I carriers [25]. The differences between studies may be associated with the studied population, considering that Santana and co-workers evaluated elderly individuals. Other factors that could explain the differences include the moment of BP measurement (only after exercise) and the type of exercise used.

Moreover, Hagberg et al. (2001) observed superior values (~10 beats) of HR maximum and cardiac output (~25%) for homozygotic II women compared with ID/DD [26]. However, these results were obtained during treadmill exercise, not after the effort as in the present study. Another possible explanation for the higher HR values after the RT session for the allele I carriers found in the present study, would be an improved autonomic adjustment as compared with allele D carriers [27]. These observations indicate that ACE gene polymorphism may present some influence on the mechanisms responsible for HR regulation.

PEH is a well-documented phenomenon in response to aerobic, resistance and water exercise [28-30]. The involved mechanisms are: a decrease of cardiac output and peripheral vascular resistance due to a lower sympathetic activity, as well as a higher activity of the kallekrein system mediating nitric oxide release [24,31,32]. In the present study, without considering the polymorphism, PEH was accompanied by a compensatory increase in HR, possible to maintain the cardiovascular homeostasis.

When evaluating data from the present study it is important to consider the reduced number of participants in each group, lack of a 24h analysis of BP, the analysis of only one gene, considering the multiple gene influence on BP, apart from the lack of other RT intensities. However, some methodological precautions were taken to minimize possible confounding issues, between them, the homogeneity of the anthropometric, cardiovascular and metabolic variables, rigid control of BP measures and RT variables, apart from the estimated sample power fixed at 85%.

Although ACE polymorphism has been associated with cardiovascular, metabolic and functional phenotypes, the discrepancy between the results of association studies with the ACE gene reveals the necessity of caution when trying to respond a macro event, such as BP and HR response to a moderate intensity RT session, especially with the analysis of only one gene, feasible of modulation by several genes. Multiple gene polymorphism analysis, including candidates such as beta 1-adrenergic receptor, angiotensin II (Ang II) Type 1 receptor (ATlR) and endothelial nitric oxide synthase (eNOS) would be of great importance. Moreover, non genetic factors may modulate BP results, such as lifestyle, nutrition, between others, obtaining verisimilitude between its pairs of only carrying the same genotype of ACE.

## Conclusion

In summary, the results of the present study revealed a possible association of ACE gene polymorphism with cardiovascular response to RT in sedentary women. DD carriers exhibit a higher effect size magnitude on PEH, while allele I carriers present a higher increase of HR after a RT session. Those individuals with a greater probability to develop cardiovascular disorders, such as DD carriers, may need different exercise intensities and volume for health prevention. These results may be used to aid RT prescription considering genetic factors.

## Competing interests

The authors declare that they have no competing interests or non-financial competing interests that may cause embarrassment to become public after the publication of the manuscript.

## Authors’ contributions

JCS: study idealization and design, data collection, writing of the introduction, results, discussion and conclusion. RAT: participation in the selection of the individuals, study idealization, data collection and methods. NMFS: participated of the statistical analysis and writing of the methods. VCS: participated in the study design, biochemical and genetic analysis, and writing of the methods. MGOK: participated in the study design, biochemical and genetic analysis, and writing of the methods. JP: study idealization and design, data collection, writing of the introduction, results, discussion and conclusion. CSGC: participated in the selection of the participants, data collection, writing of the introduction, results and conclusion. All authors read and approved the final manuscript.

## Pre-publication history

The pre-publication history for this paper can be accessed here:

http://www.biomedcentral.com/1471-2261/13/3/prepub
